# Further Insight into the Effectiveness of a Behavioral Teacher Program Targeting ADHD Symptoms Using Actigraphy, Classroom Observations and Peer Ratings

**DOI:** 10.3389/fpsyg.2017.01157

**Published:** 2017-07-11

**Authors:** Betty Veenman, Marjolein Luman, Jaap Oosterlaan

**Affiliations:** Clinical Neuropsychology Section, Faculty of Behavioural and Movement Sciences, Vrije Universiteit Amsterdam Amsterdam, Netherlands

**Keywords:** behavioral teacher program, school, ADHD, actigraphy, classroom observation, peer ratings

## Abstract

**Objective:** The Positivity and Rules program (PR program), a low-level behavioral teacher program targeting symptoms of attention-deficit/hyperactivity disorder (ADHD), has shown positive effects on teacher-rated ADHD symptoms and social functioning. This study aimed to assess whether program effects could be confirmed by instruments assessing classroom behavior other than teacher-ratings, given teachers’ involvement with the training.

**Methods:** Participants were 114 primary school children (age = 6–13) displaying ADHD symptoms in the classroom, who were randomly assigned to the treatment (*n* = 58) or control group (*n* = 65). ADHD symptoms were measured using classroom observations and actigraphy, and peer acceptance was measured using peer ratings. Intention-to-treat multilevel analyses were conducted to assess program effects.

**Results:** No beneficial program effects were found for any of the measures.

**Conclusion:** The earlier beneficial program effects on both ADHD symptoms and social functioning reported by teachers, may be explained by a change in the perception of teachers rather than changes in the child’s behavior. Other methodological explanations are also discussed, such as differences between instruments in the sensitivity to program-related changes. The current study underlines the importance of using different measures of classroom behavior to study program effects.

**ClinicalTrials.gov registration number:** NCT02518711

## Introduction

Attention-deficit/hyperactivity disorder (ADHD) is characterized by age-inappropriate, pervasive and persistent hyperactivity/impulsivity and inattention, resulting in a daily impairment in multiple settings (American Psychiatric Association, 2013). Approximately 5% of all school-aged children are affected by this disorder, which is strongly associated with multiple comorbidities (e.g., oppositional defiant disorder, conduct disorder, and anxiety disorder), and with substantial long-term risks, such as academic or occupational failure, substance abuse, and delinquent behavior (Molina et al., 2007; Polanczyk et al., 2007; Daley and Birchwood, 2010). Similar adversities, albeit in a milder form, are experienced by an additional 11% of all children, displaying ADHD symptoms without meeting full diagnostic criteria (Willcutt, 2012; Hong et al., 2014). Behavioral programs are advised as first-line treatment for ADHD, particularly for children with mild to moderate ADHD and for children with ADHD symptoms not meeting full diagnostic criteria (Pelham and Fabiano, 2008; Atkinson and Hollis, 2010). Given that many children with ADHD symptoms experience problems at school, such as academic problems, disruptive classroom behavior and teacher disobedience (Diamantopoulou et al., 2007), targeting ADHD symptoms in the school setting is an important treatment goal.

For the management of ADHD symptoms in the classroom, behavioral programs are generally preferred by teachers over medication (Pelham et al., 2007; Girio and Owens, 2009). However, most existing behavioral teacher programs are expensive and require extensive training of teachers, which can reduce long-term sustainability of such programs (Wilson et al., 2003). The authors of the current study developed the Positivity and Rules program (PR program; Veenman et al., 2016), a self-help behavioral program targeting ADHD symptoms in the classroom, which relies on a manual and can be used by teachers without additional training. An earlier study showed that the PR program improves teacher-rated ADHD symptoms and social functioning (Veenman et al., 2016). No significant effects were found on teacher-rated conduct problems and peer problems, nor did the positive effects generalize to the home setting. The teacher-rated improvements in ADHD symptoms and social behavior are important as improved behavior in the classroom could set the stage for a better teacher–child interaction, which might, in turn, improve both academic achievement and behavioral adjustment (Baker, 2006; Lassen et al., 2006).

The current study examined whether the teacher observed beneficial effects of the PR program on ADHD symptoms and social functioning could be confirmed by other measures of classroom functioning, as teachers may have been biased due to treatment involvement (Jadad and Enkin, 2008). Meta-analytic literature (Sonuga-Barke et al., 2013) suggests that effects of behavioral (parent) programs on ADHD symptoms are not corroborated by less-proximal assessments, consisting of raters not involved in treatment delivery (Sonuga-Barke et al., 2013). In order to get further insight into the effectiveness of the PR program, classroom measures other than teacher ratings were used in the current study, including classroom observations, actigraphy, and peer ratings.

Classroom observations are frequently used to assess disruptive classroom behavior and are regarded as the gold standard in research into classroom behavior (Pelham et al., 2005). A major advantage of classroom observations over questionnaires and rating scales is that behavior is measured as it occurs in the natural school setting, which is likely to enhance ecological validity (Nock and Kurtz, 2005). Many observations such as the Classroom Observation Code (COC; Abikoff and Gittelman, 1985) used in this study, are reliable and able to discriminate hyperactive children from non-hyperactive children, and there is also evidence for the sensitivity of classroom observations to the effects of behavioral and pharmacological interventions (Abikoff et al., 1980; Pelham et al., 2005). Unfortunately, few randomized controlled trials (RCTs) into the effectiveness of behavioral teacher programs for ADHD have used classroom observations (MTA Cooperative Group, 1999; Miranda et al., 2002). The two RCTs that did use classroom observations (MTA Cooperative Group, 1999; Miranda et al., 2002), either failed to report post-treatment data of the control group (Miranda et al., 2002), or did not yield any significant program effects on classroom observations while teacher-rated program improvements were found (MTA Cooperative Group, 1999).

Another example of an instrument that can be used to assess ADHD symptoms in the classroom is actigraphy. Actigraphs are wrist-worn monitors registering movement-induced accelerations used to assess daytime hyperactivity, sleep, and circadian activity rhythms, and are free from many biases associated with classroom observations or questionnaires, such as social desirability, child reactivity or halo effects (Gironda et al., 2007). Actigraphs have demonstrated higher levels of hyperactivity in hyperactive boys compared to normal controls, not only in clinical settings, but also during structured school activities (e.g., reading or mathematics, Porrino et al., 1983b; Dane et al., 2000). In fact, Porrino and colleagues were able to correctly classify 75% of the participating hyperactive boys and normal controls, using school activity assessed with actigraphs as outcome. In addition, actigraphs are sensitive to medication effects in hyperactive children (Porrino et al., 1983a; Boonstra et al., 2007). However, to our knowledge, no study has investigated the effects of a behavioral teacher program on hyperactivity using actigraphy.

Peer ratings might be used to assess program effects on classroom social functioning. While teachers using the PR program have not observed effects on peer problems, improvements have been found on teacher-rated social functioning (Veenman et al., 2016). Given these inconsistent results and the overwhelming literature indicating peer difficulties (e.g., more peer rejection and less peer acceptance) in children with ADHD symptoms (Diamantopoulou et al., 2007; Hoza, 2007), we aimed to investigate whether the PR program improved peer acceptance of children with ADHD symptoms. There are several important advantages of peer ratings. Peers observe each other’s behavior in diverse contexts (i.e., lunch room, playground), in the absence of adults, resulting in a more elaborate assessment of social functioning compared to teacher and parent ratings of social functioning (Bukowski et al., 2012). In addition, peer ratings are based on the collective knowledge of an entire classroom, thus decreasing biases compared to single source information (Surowiecki, 2004; Bukowski et al., 2012). Despite these advantages of peer ratings, the number of RCTs using peer ratings to assess effects of behavioral teacher programs targeting ADHD symptoms on social functioning is scarce and results are inconsistent (Pelham et al., 1993; Hoza et al., 2005). One study revealed significant beneficial effects of behavioral modification on peer-rated social functioning (Pelham et al., 1993). On the contrary, the Multimodal Treatment Study of children with ADHD (MTA) showed that children from all treatment groups (behavior therapy, medication, combined therapy, and community care) remained equally impaired in their peer relationships, despite superior teacher-rated social functioning in the group receiving combined therapy (Hoza et al., 2005).

The current study was designed to assess whether earlier effects of the PR program on teacher-rated ADHD symptoms and social functioning could be confirmed by other classroom measures, using actigraphy, classroom observations, and peer-ratings. Based on the teacher-reported improvements on ADHD symptoms (Veenman et al., 2016), a reduction of actigraphic hyperactivity and of classroom-observed ADHD symptoms was expected. Given the inconsistent effects of the PR program on social functioning (improvement on teacher-rated social functioning but no effects on teacher-rated peer problems) (Veenman et al., 2016), no specific hypothesis was formed for this outcome measure.

## Materials and Methods

### Participants

The study sample comprised 114 primary school children (6–13 years) displaying high levels of ADHD symptoms in the classroom (Veenman et al., 2016). Participants were randomly assigned at school level to the treatment group receiving the PR program (*n* = 58 from 44 classrooms of 30 schools; 91% male), or the control group (*n* = 56 from 43 classrooms of 34 schools; 77% male) that was allowed to receive care as usual.

**FIGURE 1:**
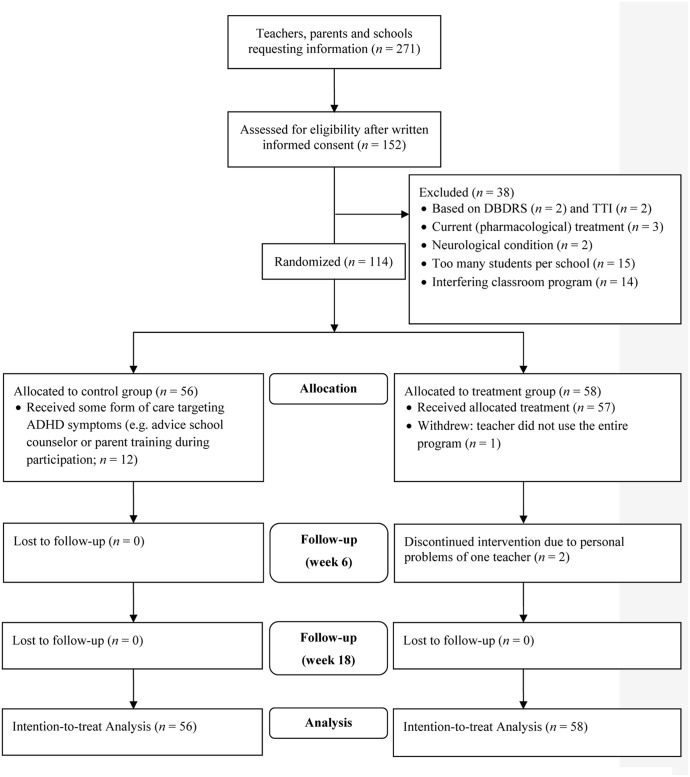
Consolidated Standards of Reporting Trials (CONSORT) flow diagram of participants during enrollment: allocation, follow-up and analysis. DBDRS = Disruptive Behavior Disorder Rating Scale; TTI = Teacher Telephone Interview. Reprinted with permission of SAGE publishing from Veenman et al. (2016).

Inclusion criteria were: (a) high levels of ADHD symptoms (>90th percentile) as reported by the child’s teacher on the Hyperactivity/Impulsivity and/or Inattention scale of the Disruptive Behavior Disorders Rating Scale (DBDRS; Pelham et al., 1992; Oosterlaan et al., 2008), and (b) at least three clinical and three subthreshold ADHD symptoms on the Teacher Telephone Interview (TTI; Holmes et al., 2004), a semi-structured interview based on Diagnostic and Statistical Manual of Mental Disorders (DSM-IV; American Psychiatric Association, 2000). Exclusion criteria were: (a) IQ < 80 estimated using a short version of the Wechsler Intelligence Scale for Children (WISC-III, including Block Design and Vocabulary; Sattler, 1992); (b) a neurological or severe physical condition interfering with daily functioning; (c) treatment for ADHD (including medication) at study entry or in the preceding 6 months, or (d) participant being enrolled in a daily behavioral teacher program or another teacher program addressing behavior or social problems at study entry or in the preceding month. The latter two exclusion criteria were used to assure assessing the isolated effects of the PR program. No more than two children per classroom and five classrooms per school were allowed to participate to limit teacher burden and to increase heterogeneity of teacher and school settings involved (Scherbaum and Ferreter, 2009). **Figure 1** displays the flowchart of participants.

### Positivity and Rules Program (Druk in de Klas)

The PR program consists of a behavioral teacher program addressing ADHD symptoms in the classroom through a teacher manual without additional expert training (see also Veenman et al., 2016). The 18-week program involves common elements of evidence-based behavioral programs (e.g., Summer Treatment Program; MTA Cooperative Group, 1999) such as psycho-education for the teacher, and classroom behavior management strategies comprising both antecedent and consequent behavioral techniques (e.g., positively formulated classroom rules, effective teacher instructions, universal reward system and time-out) (Pelham and Fabiano, 2008). While these behavioral techniques were administered to the entire classroom (i.e., to the children with ADHD symptoms and to all of their classmates), an individual program with three intensity levels was available for children with ADHD symptoms that consisted of a Daily Report Card. Although several techniques will be familiar to many teachers, the manual instructs teachers on how to systematically and adequately implement all program elements by providing detailed practical instructions on implementation (including work sheets, flow diagram and agenda). For more information about the PR program, see our previous study (Veenman et al., 2016).

### Outcome Measures

Actigraphy and classroom observations were used to assess the effects of the PR program on ADHD symptoms in the classroom, while peer ratings were used to assess whether peer acceptance improved after use of the program.

#### Actigraphy

Hyperactivity at school was measured by small (37 mm × 29 mm × 10 mm), light-weight (16 g) actigraphs (Actiwatch 4, Philips Respironics, Murrysville, PA, United States), detecting the highest movement-induced accelerations (0.5–7.0 Hz) during 15-s intervals, generating a transient voltage signal proportionate to the acceleration rate (Chen and Bassett, 2005; Cambridge Neurotechnology, 2008). Good inter-unit reliability (concordance between actigraphs worn at the same body parts) and convergent validity with a validated three-dimensional motion-tracking system has been established (Gironda et al., 2007). Intra-unit reliability of the actigraphs used in this study, calculated as the correlation of average activity counts per minute of each actigraph between all school days, was good (Cronbach’s α = 0.77).

The actigraphs were worn on the non-dominant hand during seven consecutive days, of which the five school days were used to assess hyperactivity at school. As school hours are similar across schools in the Netherlands, the selected hours for data-analysis were kept similar for every child: 9:00–15:00 on Monday, Tuesday, Thursday and Friday, and 9:00–12:00 on Wednesday. For Grade 1 and 2 (40% of all children), data from Friday afternoon (12:00–15:00) were excluded because at most schools, school ends at 12.00 on Fridays in these grades. Data for days on which scores were missing (e.g., due to technical failure or participant non-adherence) or special non-academic events (e.g., holiday, school trip or illness) were also excluded. This resulted in 25.5% missing data, which is similar to other studies using actigraphy (Acebo et al., 1999; Ustinov and Lichstein, 2011). Average activity counts per minute during the school hours across five school days served as the dependent measure and was calculated using Respironics Actiware software (Version 5.71.0; Philips Respironics, Murrysville, PA, United States).

#### Classroom Observation

Children were observed in the classroom during structured academic lessons (i.e., teacher instructions or individual work during academic tasks, such as mathematics and writing) using the COC (Abikoff and Gittelman, 1985). The COC was administered as in the original form (1985), during two blocks of 8 min, divided into 15-s intervals. As the dependent measure in our analyses, the observed behavior rate (i.e., mean percentage of intervals in which behavior occurred) of Interference (Interference and Interference to Teacher measuring impulsivity), Off-Task (measuring inattention) and Gross Motor-all (Gross Motor-standing and –vigorous measuring hyperactivity) were summed into one ADHD-composite score as done previously in other treatment studies (Klein and Abikoff, 1997; Abikoff et al., 2004).

Participants were observed by pairs of two trained raters who were not blind to treatment allocation of the child due to visibility of program elements in the classroom. Observers received an elaborate training that consisted of didactic lessons and scoring *in vivo*. The COC has been shown to discriminate children with ADHD from typically developing peers (Abikoff et al., 2002), and has been shown sensitive to treatment effects (Abikoff et al., 2004). High interrater reliability has been reported for all categories of the COC (φ = 0.80–1.00, *M* = 0.91; Abikoff et al., 2002).

#### Peer Acceptance

The child‘s social acceptance by peers in the classroom was measured through peer ratings, requiring each student to indicate the likability of all classmates on a 5-point Likert scale from 1 (dislike very much) to 5 (like very much; Pelham et al., 2000). The participant’s average peer score was used as dependent measure, by summing the scores of all classmates and then dividing this total score by the number of children in the classroom. Test–retest reliability of this measure was high (*r* = 0.72) and validity of peer ratings as measure of social acceptance is adequate when compared to teacher-rated social acceptance (Maassen et al., 2000; Wu et al., 2001).

#### Implementation Fidelity

A teacher questionnaire (available on request) was administered at the end of each week to acquire an indication of implementation fidelity. This checklist contains 13 items on a 3-point Likert scale, requiring teachers in the treatment group to indicate whether and to what extent they used each of the program elements during that week (0 = not used or inadequate use, 1 = adequate use, and 2 = good use). The average item score was calculated based on all weekly checklists. Internal consistency for this measure was acceptable (Cronbach’s α = 0.67).

### Procedure

This study was conducted in the Netherlands between September 2011 and July 2014. Teachers and parents were recruited through educational consultant associations, the national parent association for children with developmental problems, and the study’s website. Teachers and parents showing interest in participating in the study received an information letter explaining the research aim and responsibilities of all parties involved. In case teachers were interested in participating, they enlisted one or two children displaying ADHD symptoms in their classroom. Written informed consent was obtained from all parents, teachers, and children older than 11 years. Children were screened for eligibility by the first author BV. ML, not in contact with any of the children, was responsible for the subsequent computer-generated randomization (replacement randomization, without stratification) to allocate children to the treatment or control group. Although teachers in the treatment group used the universal program in the entire classroom, the effectiveness of the PR program was only investigated for the participating children displaying ADHD symptoms. Dependent variables were measured at baseline (T_o_; week 0), 6 weeks after starting the PR program (T_1_), and after 18 weeks at the end of the program (T_2_). Financial compensation was given to all participating teachers (control group: €50; treatment group: €125). This study was carried out in accordance with the recommendations of the medical ethical committee of the Vrije Universiteit Amsterdam with written informed consent from all subjects. All subjects gave written informed consent in accordance with the Declaration of Helsinki. The protocol was approved by the medical ethical committee of the Vrije Universiteit Amsterdam (reference number 2011/196).

### Statistical Analyses

Sample size estimation was performed using the software Optimal Design (Liu, Spybrook, Congdon, Martinez, and Raudenbush, 2005–2011). Assuming a moderate effect size of 0.50, a sample size of 116 was calculated to be sufficient for a repeated-measures multilevel analysis with a maximum of two participants per classroom, using an alpha of 0.05, a power of 80% and a intraclass correlation of 0.10.

To evaluate the effects of the PR program, multilevel analyses were conducted using the Statistical Package for the Social Sciences (SPSS; IBM Corp, 2011). All randomized subjects participated in the intention-to-treat analyses, regardless of the amount of missing data. For none of the measures, the percentage of missing data exceeded 5% (5.0% for classroom observations and for teacher ratings on implementation fidelity, and 4.7% for peer ratings), except for actigraphy for which 25.5% of data were missing (see subheading Actigraphy). Four hierarchical levels were used: observations at level 1 were nested within students (level 2), level 2 was nested within classrooms (level 3), and level 3 was nested within schools (level 4; Heck et al., 2013). Group was used as fixed factor with control group as reference group. Time was used as fixed factor and was expressed in number of weeks (0, 6, and 18 weeks for T_0_, T_1_, and T_2_, respectively). The interaction between group and time was used to evaluate whether behavior of children in the treatment group improved more over time compared to children in the control group. Analyses controlled for baseline levels of the dependent variables and for gender differences (there were more boys in the treatment group; see Results), inserting gender as fixed factor (with boys as reference group). The Likelihood Ratio Test and Akaike’s Information Criteria (AIC) were used to determine whether random time slopes at student-, classroom- or school-level needed to be included for providing better model fit (Heck et al., 2013). Alpha-level was set at 0.05.

## Results

Sample characteristics of the treatment and control group are displayed in **Table 1**. No group differences were found on age, IQ, socioeconomic status, race, the presence of ADHD or other psychiatric diagnoses, parent- and teacher-rated ADHD symptoms assessed with DBDRS, and inattention and combined ADHD symptoms assessed with the TTI (*p*-values > 0.261). However, children in the treatment group were more likely to be boys [χ^2^(1) = 4.56, *p* = 0.033], and received higher ratings of hyperactive/impulsive behavior on the TTI [*t*(112) = -2.32, *p* = 0.022]. All subsequent analyses controlled for gender differences and controlled for baseline levels of the dependent variable (see below).

**Table 1:** Demographic and clinical characteristics of participants (*N* = 114) in treatment and control group.

	Treatment	Control)
	group (*n* = 58)	group (*n* = 56)
**Demographic Characteristics**		
Age (years)	8.48 (1.85)	8.25 (1.97)
Gender (% male)^∗^	91% (*n* = 53)	77% (*n* = 43)
IQ	104.02 (11.34)	100.21 (10.41)
SES^a^	3.37 (0.67)	3.24 (0.95)
Race (% Caucasian)	86% (*n* = 50)	82% (*n* = 46)
ADHD diagnosis	10% (*n* = 6)	9% (*n* = 5)
Other psychiatric diagnosis	2% (*n* = 1; CD)	2% (*n* = 1; PDD-NOS)
**Parent DBDRS**		
Inattention	11.71 (5.23)	10.77 (5.57)
Hyperactivity/Impulsivity	11.79 (5.42)	10.47 (5.49)
ODD	5.88 (3.84)	4.93 (4.00)
CD	0.86 (1.31)	0.90 (1.40)
**Teacher DBDRS**		
Inattention	14.63 (5.26)	14.90 (5.83)
Hyperactivity/Impulsivity	15.67 (5.35)	15.03 (6.23)
ODD	6.77 (4.75)	5.95 (4.75)
CD	1.43 (1.65)	1.57 (1.90)
**TTI**		
Inattention	12.50 (6.12)	12.45 (5.34)
Hyperactivity/Impulsivity^∗^	15.62 (6.03)	12.88 (5.78)
Combined	28.12 (8.97)	25.33 (8.34)
**Outcome measures**		
Actigraphy (AC/min)	734.24 (223.54)	730.79 (188.16)
Classroom-observed ADHD symptoms	25.33 (19.26)	21.59 (18.64)
Peer acceptance	3.17 (0.68)	3.24 (0.71)


**M* and *SD*s are depicted unless stated otherwise. AC/min = Activity Counts per minute; ADHD = Attention-Deficit Hyperactivity Disorder; CD = Conduct Disorder; DBDRS = Disruptive Behavior Disorder Rating Scale; ODD = Oppositional Defiant Disorder; PDD-NOS = Pervasive Developmental Disorder-Not Otherwise Specified; SES = Socioeconomic Status; TTI = Teacher Telephone Interview. ^a^SES was measured by parental educational level (average of both parents) through an adapted version of the Dutch classification system (1 = primary education, 2 = secondary vocational education, 3 = secondary general education, 4 = undergraduate school, 5 = graduate school; Verhage, 1983). ^∗^ < 0.05. Adapted with permission of SAGE publishing from Veenman et al. (2016).*

### Implementation Fidelity and Teacher Satisfaction

Most teachers (81%) reported adequate or good implementation of the universal reward system during the entire program, with the remaining 19% reporting inadequate implementation in 1 or 2 weeks during the course of the entire 18 weeks. Teachers reported to have used all elements of the DRC adequately most of the 18 weeks (*M* = 78% of the time; *SD* = 23%). The helpdesk was consulted by 30% of the teachers and mainly received questions regarding the use of the time-out and the reward system for the entire classroom or individual children. Satisfaction rate was high: 98% of the teachers intended to use the entire program or most important program elements in the future (71 and 27%, respectively).

### Effectiveness PR Program

Multilevel results are depicted in **Table 2**. For all outcomes, four-level models were reduced to three-level models as intercept variance was zero at school level.

**Table 2:** Multilevel results on ADHD symptoms and peer acceptance.

	ADHD symptoms (observation)			Hyperactivity (actigraph)^a^			Peer acceptance		
			
	Coefficient	*SE*	*p*-value	Coefficient	*SE*	*p*-value	Coefficient	*SE*	*p*-value
**Fixed effects**								
Constant	14.649	3.833	<0.001	326.929	55.046	<0.001	0.465	0.120	<0.001
Baseline level of DV	0.278	0.069	<0.001	0.527	0.060	<0.001	0.830	0.033	<0.001
Gender (Boys = 0)	-4.435	4.555	0.215	-21.745	33.342	0.515	-0.024	0.059	0.687
Group (Time = 0)^b^	1.635	4.837	0.736	16.919	46.423	0.716	0.139	0.077	0.071
Time	-0.140	0.236	0.555	0.699	2.318	0.763	0.012	0.004	0.008
Group^∗^Time	0.133	0.337	0.639	0.565	3.285	0.864	-0.002	0.006	0.760
**Random effects**^c^								
$\sigma_{u0}^{2}$ (classroom)	40.675	33.813		-	-		0.052	0.011	
$\sigma_{u0}^{2}$ (student)	30.645	37.966		4938.676	2558.918		0.086	0.014	
σ_u01_ (student)	-	-		-	-		-0.008	0.001	
$\sigma_{u1}^{2}$ (student)	-	-		-	-		0.0001	0.0001	
$\sigma_{e}^{2}$	211.746	29.281		17830.428	2724.235		0.051	0.007	


*ADHD, Attention-Deficit Hyperactivity Disorder; DV, Dependent Variable. $\sigma_{u0}^{2}$ = Random intercept; σ_*u01*_ = Covariance between random intercept and random slope; $\sigma_{u1}^{2}$ = Random slope.^*a*^Average hyperactivity at school in Activity Counts per minute.^*b*^The fixed group effect represents group differences at baseline. The control group was used as reference group.^*c*^Only the random intercepts and random time slopes that were part of any of the final models are depicted in this Table.*

#### Actigraphic Hyperactivity

At baseline, children in the treatment group showed similar levels of hyperactivity as assessed by actigraphy compared to controls (*p* = 0.932). Hyperactivity did not change over time (*p* = 0.763; see **Table 2** for coefficients), nor were time slopes significantly different for children in the treatment group and control group (*p* = 0.864) when controlling for baseline hyperactivity and gender differences.

#### Classroom-Observed ADHD Symptoms

Results on the classroom-observed ADHD-composite showed that children in the treatment and control group displayed similar levels of ADHD symptoms at baseline (*p* = 0.300). ADHD problems did not change over time (*p* = 0.555), nor were time slopes significantly different for children in the treatment group and control group (*p* = 0.693) when controlling for baseline ADHD symptoms and gender differences.

#### Peer Acceptance

Children in the treatment and control group displayed similar levels of peer acceptance at baseline (*p* = 0.628). Peer acceptance improved over time (*p* = 0.008), but this improvement was similar for children in the treatment group and control group (*p* = 0.760) when controlling for peer acceptance at baseline and gender differences.

#### Correlations between Instruments

To study the inconsistency between the positive effects of the PR program on teacher ratings and the classroom measures showing no effects in the current study, exploratory correlational analyses were performed at baseline between ADHD measures of this study and of our previous study (Veenman et al., 2016). Hence, classroom-observed ADHD symptoms and actigraphic hyperactivity were both correlated with teacher-rated ADHD symptoms as measured by the Strengths and Weaknesses of ADHD-symptoms and Normal Behavior (SWAN; Swanson et al., 2006). Similarly, peer acceptance was correlated with teacher ratings assessing peer problems (i.e., Peer Problems Scale of the Strengths and Weaknesses Questionnaire, SDQ; Van Widenfelt et al., 2003) and general social functioning (i.e., Social Skills Rating Scale; Gresham and Elliott, 1990). Results showed that both classroom-observed ADHD symptoms and actigraphic hyperactivity did not significantly correlate with teacher-rated ADHD symptoms (SWAN Total score; *r* = -0.12, *p* = 0.230 and *r* = -0.18, *p* = 0.075, respectively). As actigraphy measures hyperactivity rather than general ADHD symptoms, the correlation between actigraphic hyperactivity and the SWAN Hyperactivity/Impulsivity scale (teacher version) was also assessed, yielding a significant, small-to-moderate correlation (*r* = -0.25, *p* = 0.012, *R*^2^ = 0.06). Results revealed a large and significant correlation between peer acceptance and teacher-rated peer problems (i.e., Peer Problems Scale of the SDQ; *r* = -0.53, *p* < 0.001, *R*^2^ = 0.28), and a medium to large correlation between peer acceptance and teacher-rated general social functioning (*r* = 0.37, *p* < 0.001, *R*^2^ = 0.14).

## Discussion

This study aimed to assess whether earlier reported teacher-rated effects of the PR program on ADHD symptoms and social functioning (Veenman et al., 2016) could be confirmed by other classroom measures of ADHD symptoms and social functioning, using actigraphy, classroom observations and peer ratings. For none of these measures beneficial effects of the PR program were found. The current findings are in line with literature indicating that positive effects of behavioral (parent) programs on ADHD symptoms are not confirmed by less-proximal instruments rather than parents who were involved in treatment delivery (MTA Cooperative Group, 1999; Sonuga-Barke et al., 2013; Daley et al., 2014). With regard to peer acceptance, improvement was unrelated to use of the PR program. This result is in accordance with earlier findings showing no beneficial effects of behavioral treatments and treatments combining behavioral and medication therapy on peer problems, despite improvements on teacher-rated social functioning (Pelham et al., 1993; Hoza et al., 2005; Veenman et al., 2016).

Our results suggest that the teacher-reported effects might reflect a change in perception of the teacher regarding the child’s functioning, rather than actual behavioral changes within a child, which could not be captured by the other measures used in the current study (i.e., classroom observations, actigraphy or peer ratings). This change in teachers’ perception could be explained by teachers being biased due to their investment in the program and subsequent positive treatment expectations (Sonuga-Barke et al., 2013). An alternative explanation is that teachers show increased tolerance or coping with ADHD symptoms (Daley et al., 2014), for example due to the psycho-education, which has been found to increase positive attitudes and behavior toward individuals with ADHD (Nussey et al., 2013).

One partial explanation for the absence of significant program effects in the current study, might be related to the degree of severity of our sample. Children in our study all showed high levels of ADHD symptoms, but children receiving treatment for ADHD were excluded. As a result, only 10% were diagnosed with ADHD and 2% had a comorbid psychiatric diagnosis. Consequently, the room for improvement in terms of ADHD symptoms was lower in our sample compared to clinical ADHD samples, which may have added to the non-significant treatment effects for the current dependent variables.

Another methodological explanation for the lack of significant program effects on the ADHD measures should also be considered. The low correlations between teacher-rated ADHD measure in our previous study and the ADHD measures of the current study (classroom observation and actigraphy) suggest that these measures pertain to different aspects of the child’s behavior. With regard to the wrist-worn actigraphs, this measure mainly registers upper limb movements (Kumahara et al., 2004), whereas teacher-rated Hyperactivity/Impulsivity concerns more than just upper-limb hyperactivity and also involves impulsivity, such as difficulty awaiting turns. Another aspect inherent to actigraphic measurements is their inability to distinguish inappropriate hyperactivity from appropriate activity, such as raising hands before asking a question. This illustrates the difficulty of using actigraphs to assess effects of behavioral programs targeting hyperactivity. Waist- or ankle-worn actigraphs could be a more appropriate measure of general activity (Gironda et al., 2007), and might therefore be more suitable to investigate whether behavioral programs reduce hyperactivity in the classroom. With regard to the classroom observations, the validity might have been limited by the narrow time frame within which observations took place (2 × 8 min in a structured setting) compared to teacher ratings (SWAN) that were based on behavior shown in the last 4 weeks (Sonuga-Barke et al., 2013). The absence of program effects on the current less-biased ADHD measures despite teacher-rated improvements of the PR program, may thus be related to the different behavioral aspects measured by these instruments.

The lack of program effects on peer acceptance in our current study is in line with the lack of effects of the PR program on teacher-rated peer problems and could have been expected as the PR program focused on improving ADHD symptoms rather than improving peer problems (Veenman et al., 2016). These findings are also in accordance with the MTA study, in which sociometric outcomes were resistant to both medication and behavioral therapy (Hoza et al., 2005). The current results suggest that targeting child behavior is insufficient to change peer status, particularly in short interventions such as this 18-week program. Peer rejection is highly stable and stability of negative peer perception is not only explained by the behavioral problems of the rejected child itself, but also by peer group influences such as social devaluation, exclusionary behavior and reputational bias (Mikami and Normand, 2015). For example, peers can socially devalue children with ADHD symptoms because they are dissimilar from other classmates and are viewed as being responsible for their own actions (Hinshaw, 2005). Peers can also display exclusionary behavior to disliked children (e.g., saying mean things about the child within earshot or spreading lies), thus exacerbating peer problems and fueling peer rejection by other classmates (Perry et al., 1988; Reuland and Mikami, 2014). Besides that, peers can attribute behavior improvement of rejected children to external and unstable factors (Hymel, 1986) and are likely to interpret ambiguous behavior of disliked children as hostile, while evaluating similar behavior of beloved children more positively (Peets et al., 2008). Hence, improving the behavior of children with ADHD symptoms is probably insufficient to reduce peer rejection. Teachers should engage in strategies that directly focus on changing peer culture, such as the intervention Making Socially Accepting Inclusive Classrooms (MOSAIC; Mikami et al., 2013), in order to improve peer acceptance.

It could be argued that additional expert involvement could have improved the effectiveness of the PR program, but literature on this topic is inconsistent. While a review on the effectiveness of self-help interventions for parents of children with behavior problems suggests that adding minimal levels of therapeutic support improves child outcomes (O’Brien and Daley, 2011), another meta-analytic study suggests that self-help parenting interventions are equally effective compared to therapist-led parenting interventions (Tarver et al., 2014). So far, no studies have investigated the added effects of therapist support to low-level teacher programs. More research is needed to assess whether the effectiveness of the PR program can be improved through therapist involvement.

A few limitations of our study should be noted. First, observers were not blind to treatment allocation due to the visibility of some program elements within the classroom. Although this could have resulted in observer’s bias, results were probably not influenced because no program-related effects in favor of the treatment group were found. Another limitation of the PR program is that implementation fidelity of teachers was measured by self-report instead of independent classroom observations (see also Veenman et al., 2016). Third, treatment expectations were not assessed, which prevented us from investigating whether the discrepancy between current results and earlier significant teacher-reported effects could be explained by an expectation effect of teachers in the treatment group.

## Conclusion

This study is unique in its use of classroom observations, actigraphy and peer ratings to investigate whether earlier teacher-reported effects of the PR teacher program on ADHD symptoms and social functioning could be confirmed by other classroom measures. Results showed that improvements in teacher-rated ADHD symptoms were not confirmed by either actigraphy or by classroom observations. Possibly, the PR program improves teachers’ perception of a child’s behavior, rather than the child’s behavior. However, the possibility that the current measures assess different aspects of classroom behavior than teacher ratings should not be ruled out either, nor the potential influence of other methodological explanations (e.g., limited sensitivity to program-related improvements on ADHD symptoms). Either way, improving teachers’ perception may be an important step to enhance teacher–child interaction, which is in turn essential for the child’s behavioral, social and academic functioning at school and thus for future success in life (Stuhlman and Pianta, 2002; Baker, 2006). Future studies should thus assess the effect of the PR program on teacher–child interaction.

## Author Contributions

BV: Data collection, statistical analyses, literature review, writing entire manuscript. ML and JO: Article revision.

## Conflict of Interest Statement

The authors declare that the research was conducted in the absence of any commercial or financial relationships that could be construed as a potential conflict of interest.
